# BNT162b2 COVID-19 vaccination elicited protective robust immune responses in pediatric patients with inborn errors of metabolism

**DOI:** 10.3389/fimmu.2022.1082192

**Published:** 2023-01-05

**Authors:** Tanyel Zubarioglu, Harika Oyku Dinc, Duhan Hopurcuoglu, Ruveyda Gulmez, Esma Uygur, Gizem Yilmaz, Saffa Ahmadzada, Gozde Uzunyayla-Inci, Ece Oge-Enver, Ertugrul Kiykim, Bekir Kocazeybek, Cigdem Aktuglu-Zeybek

**Affiliations:** ^1^ Department of Pediatric Nutrition and Metabolism, Cerrahpasa Medical Faculty, Istanbul University-Cerrahpasa, Istanbul, Türkiye; ^2^ Department of Pharmaceutical Microbiology, Faculty of Pharmacy, Bezmialem Vakıf University, Istanbul, Türkiye; ^3^ Department of Pediatric Nephrology, Cerrahpasa Medical Faculty, Istanbul University-Cerrahpasa, Istanbul, Türkiye; ^4^ Department of Pediatrics, Istanbul University-Cerrahpasa, Cerrahpasa Medical Faculty, Istanbul, Türkiye; ^5^ Department of Medical Microbiology, Cerrahpasa Medical Faculty, Istanbul University-Cerrahpasa, Istanbul, Türkiye

**Keywords:** BNT162b2, inborn errors of metabolism, humoral immune response, neutralizing antibody, children

## Abstract

**Introduction:**

SARS-CoV-2 infection can lead to a life-threatening acute metabolic decompensation in children with inborn errors of metabolism (IEM), so vaccination is mandatory. However, IEMs can also impair innate or adaptive immunity, and the impact of these immune system alterations on immunogenicity and vaccine efficacy is still unknown. Here, we investigated humoral immune responses to the BNT162b2 mRNA COVID-19 vaccine and clinical outcomes in pediatric IEM patients.

**Methods:**

Fifteen patients between 12-18 years of age with a confirmed diagnosis of IEM, and received BNT162b2 were enrolled to the study. Patients with an anti-SARS-CoV-2 IgG concentration >50 AU/mL before vaccination were defined as “COVID-19 recovered” whereas patients with undetectable anti-SARS-CoV-2 IgG concentration were defined as “COVID-19 naïve”. Anti-SARS-CoV-2 Immunoglobulin G (IgG) and SARS-CoV-2 neutralizing antibody (nAb) titers were measured to assess humoral immune response.

**Results:**

Anti-SARS-CoV-2 IgG titers and nAb IH% increased significantly after the first dose. The increase in antibody titers after first and second vaccination remained significant in COVID-19 naïve patients. Complete anti-SARS-CoV-2 IgG seropositivity and nAb IH% positivity was observed in all patients after the second dose. Vaccination appears to be clinically effective in IEM patients, as none of the patients had COVID-19 infection within six months of the last vaccination.

**Discussion:**

Humoral immune response after two doses of BNT162b2 in pediatric IEM patients was adequate and the immune response was not different from that of healthy individuals.

## Introduction

Since its declaration as a pandemic on March 11, 2020; Coronavirus Disease-19 (COVID-19) has caused high morbidity and mortality worldwide, affecting adults more severely than children. Apart from well-known comorbidities such as obesity, hypertension, heart disease, diabetes, and chronic kidney disease, in which patients are at significantly higher risk of a severe COVID-19 infection; disease progression can be challenging in pediatric patients with inborn errors of metabolism (IEM) (1). According to the medical literature, children with IEM are considered to be more vulnerable because SARS-CoV-2 infection can lead to severe and even life-threatening acute metabolic decompensation, especially in intoxication and energy metabolism disorders (2–6). In conclusion, vaccination against COVID-19 has an important role not only in preventing COVID-19 infection and transmission, but also in controlling metabolic homeostasis.

There are some special safety and efficacy considerations in the general vaccine recommendations for patients with IEM. Inborn errors of metabolism can affect innate or adaptive immunity, or both, and can present different types of immune dysregulation. A primary immunodeficiency may be the cardinal feature of an IEM, or some degree of immune deficiency may contribute to the clinical spectrum as the disease progresses (7–9). However, the impact of immune system alterations on immunogenicity and vaccine efficacy in IEM is limited (8, 10–12). The BNT162b2 mRNA COVID-19 vaccine (Pfizer-BioNTech^®^) is used worldwide and has been shown to be safe, immunogenic, and protective in children and adolescents during the pandemic (13). To date, there are no data on the immune response elicited by COVID-19 vaccines in children with IEM.

The aim of this study is to investigate humoral immune responses to the BNT162b2 mRNA COVID-19 vaccine in pediatric IEM patients by measuring anti-SARS-CoV-2 immunoglobulin G (IgG) and SARS-CoV-2 neutralizing antibodies (nAb) and assessing clinical outcomes.

## Materials and methods

### Study design and participants

This prospective, single-center case-control study was conducted at Istanbul University-Cerrahpasa, Cerrahpasa Medical Faculty, Pediatric Nutrition and Metabolism Unit between September 2021 and May 2022. Following the announcement of Turkey’s Ministry of Health about COVID-19 vaccination in children aged 12-18, IEM patients were informed about COVID-19 vaccines available in Turkey; CoronaVac (Sinovac Life Sciences, Beijing, China) and BNT162b2. Two doses of vaccination, one month apart, were recommended. Patients who have been regularly followed up by Pediatric Nutrition and Metabolism outpatients’ clinic were enrolled if they met the following inclusion criteria: i) ≥12 to <18 years of age, ii) having a biochemically and/or molecularly confirmed diagnosis of IEM, iii) received BNT162b2. Control group consisted of 15 age and sex-matched healthy volunteers without a history of acute and/or chronic infection.

Regarding the assessment of the humoral immune response to SARS-CoV-2 vaccine in the patient group, blood samples were collected before the first dose and one month after each vaccination date. In the control group, a single sampling was performed one month after the second dose of vaccination. All samples were stored at –20 °C until assayed.

Demographic, clinical, and biochemical findings of the IEM patients were reported in detail. Regarding COVID-19 vaccination; vaccination dates and the number of vaccine doses for each patient were also recorded.

This study was designed in accordance with the current revision of the Helsinki declaration and was approved by the local Ethical Committee (E-83045809.604.01.01-370424). Informed consent was obtained from each participant.

### Assessment of humoral immune response to SARS-CoV-2 vaccine

#### SARS-CoV-2 quantitative IgG test

Determination of the quantity of nAb against the receptor-binding region (RBD) of the spike protein (S1) of SARS-CoV-2 was made by SARS-CoV-2 IgG test (ARCHITECT IgG II Quant test, Abbott, USA). It was performed by the chemiluminescent microparticle immunoassay (CMIA) method. The sera results were evaluated as Arbitrary Unit/mL (AU/mL) according to the manufacturer’s cut-off value. Results were multiplied by a correlation coefficient of 0.142 and converted to “Binding Antibody Units (BAU/mL)” in the WHO’s International Standard for Anti-SARS-CoV-2 immunoglobulin (14). Concentrations above 50 AU/mL or 7.1 BAU/mL were evaluated as positive. While this test was 100% compatible with the plaque reduction neutralization test (PRNT), a concentration of 1050 AU/mL was associated with a 1:80 dilution of PRNT (15).

#### SARS-CoV-2 surrogate neutralization test

Detection of the nAb that prevents viral SARS-CoV-2 S1-RBD from binding to the ACE2 receptors of human cells was made by SARS-CoV-2 surrogate neutralization test. It was performed by competitive ELISA method (SARS-CoV-2 NeutraLISA, Euroimmun, Lübeck, Germany). Subtracting the optical density of the patient sample from the ratio of the optical density of the blank determined the neutralization capacity. The sera results were evaluated as percent inhibition (IH%). nAb IH% results were interpreted following the manufacturer’s instructions as i)IH% of < 20% was evaluated as negative, ii) IH% of 20%–35% as a breakpoint, iii) IH% of ≥35% as positive. The test is 98.6% compatible with the PRNT (16).

#### Assessment of a current COVID-19 infection prior to vaccination

Before vaccine administration, a SARS-CoV-2 reverse transcriptase–polymerase chain reaction (RT-PCR) test was performed on all participants to exclude a current COVID-19 infection. Patients with a negative SARS-CoV-2 RT-PCR result received a COVID-19 vaccine. Regarding the asymptomatic cases, an initial anti-SARS-CoV-2 IgG measurement was performed in the patients’ group. Patients with an anti-SARS-CoV-2 IgG concentration above 50 AU/mL or 7.1 BAU/mL before vaccination were defined as “COVID-19 recovered” whereas patients with undetectable anti-SARS-CoV-2 IgG concentration were defined as “COVID-19 naïve”.

#### Assessment of clinical response to COVID-19 vaccination

Six months after the last dose of the COVID-19 vaccine, patients were interviewed by phone calls and surveyed about whether they had a COVID-19 infection after vaccination. In case of a history of COVID-19 infection, the severity of the disease was determined by questioning the clinical findings, need for oxygen support, hospitalization, and intensive care unit admission. COVID-19 severity classification was made according to the criteria described by Dong et al. (17).

#### Statistical analyses

Statistical analyses were performed using Statistical Package for Social Sciences version 21.0 (SPSS Inc., Chicago, IL, USA). Continuous variables were displayed as mean (standard deviation) and median (25th; 75th percentiles) according to distribution. The normal distribution of data was evaluated with a Kolmogorov–Smirnov test. Non-normal distributed data were analyzed by Mann Whitney U and Kruskal Wallis tests. Analysis of the normally distributed categorical variables was made by paired sample t-test. In the case of a non-normal distribution, data were analyzed using the Wilcoxon test. The correlation between anti-SARS-CoV-2 IgG titers and nAb IH% values was analyzed using the Spearman correlation test. A value of p < 0.05 was considered statistically significant.

## Results

Fifteen patients and 15 age and sex-matched healthy volunteers were included in this study. The mean age of the patients and the controls was 14.1(1.7) and 14.8 (2) years, respectively. According to classification of IEM, nine patients (60%) were diagnosed with amino acid metabolism disorder; three (20%) with fatty acid and ketone body metabolism disorder; two (13.3%) with carbohydrate metabolism disorder and one (6.7%) with lipid metabolism disorder (18).

All patients and the controls received two doses of BNT162b2 at a one-month interval. Eight patients (53.3%) were found to be COVID-19 recovered and seven were (46.7%) COVID-19 naïve prior to vaccination according to initial anti-SARS-CoV-2 IgG concentrations.

**Figure 1:**
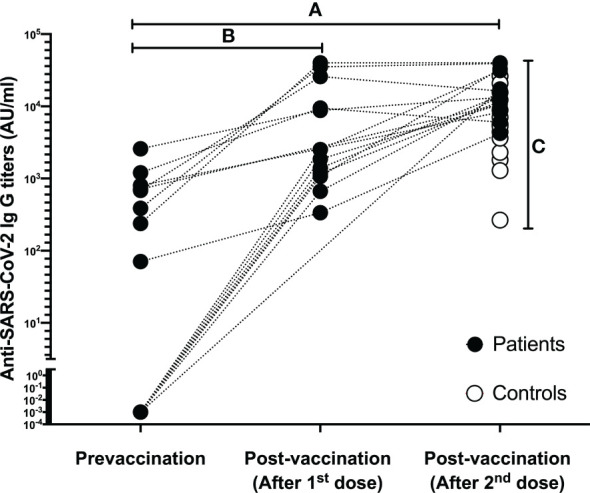
Data regarding the Anti-SARS-CoV-2 IgG titers of each individual in both patient and control group. **(A)** Complete Anti-SARS-CoV-2 IgG seropositivity was determined in patients after two doses of BNT162b2 vaccination; **(B)** Anti-SARS-CoV-2 IgG titers increased significantly after the first dose in patients who were sampled after the first vaccination; **(C)** After two doses of BNT162b2 vaccination, complete Anti-SARS-CoV-2 IgG seropositivity was also detected in the controls, and the final antibody titers were higher in favor of the patient group.

Demographic data and characteristics of the patient and control groups are given in **Table 1**, Anti-SARS-CoV-2 IgG titers before and after vaccination are given in **Figure 1**.

**Table 1:** Demographic data and characteristics of patients and controls.

Patients with IEM (n=15)								Controls (n=15)		
14.1 ± 1.7 years								14.8 ± 2.0 years		
N.	Age (y)	Sex	IEM subgroup	Mutation	Protein change	Gene affected	N	Age (y)	Sex	
1	12	M	Alkaptonuria	c.808G>A	p.G270R	HGD	1	13	M
2	15	F	Citrullinemia type 1	c.1085G>T	p.Gly362V	ASS1	2	15	F
3	14	M	Primary carnitine deficiency	c.67_69delTTC	p.delF23	SLC22A5	3	17	F
4	15	M	Lpin 1 deficiency	c.2285delC	p.Ser762Leufs*36	LPIN1	4	14	M
5	17	F	Isovaleric acidemia	c.881C>T	p.P294L	IVD	5	12	M
6	15	M	Propionic acidemia	c.1100A>T	p.Asp367Val	PCCB	6	13	F
7	13	F	Citrullinemia type 1	c.571G>A/ c.865delA	p.E191K(GAG>AAG)/ p.I289SfsX6	ASS1	7	14	F
8	12	F	Ornithine transcarbamylase deficiency	c.145A>C	p.Thr49Pro	OTC	8	17	M
9	12	F	Phenylketonuria	c.441+5G>T/ c.1066-11G>A	IVS4+5G>T/IVS10-11G>A	PAH	9	13	M
10	15	F	CPT2 deficiency	c.338C>T	p.Ser113Leu	CPT2	10	14	M
11	12	M	Hereditary tyrosinemia type 1	c.1062+5G>A	IVS12+5G>A	FAH	11	12	M
12	13	M	Maple syrup urine disease	c.773_774delGCi	p.Cys258Ter	BCKDHA	12	17	F
13	14	F	Glycogen storage disorder type 1b	c.365G>A	p.G122E	SLC37A4	13	17	F
14	16	F	Multiple acyl-CoA dehydrogenase deficiency	c.1130T>C/ c.731T>C	p.Leu377Pro/p.Phe244Ser	ETFDH	14	17	F
15	17	E	Glycogen storage disorder type 1b	c.365G>A	p.G122E	SLC37A4	15	17	F

IEM, inborn errors of metabolism; N, number; y, years; CPT2, carnitine palmitoyltransferase 2.

### Humoral immune response of the study population to SARS-CoV-2 following the second dose of BNT162b2 vaccination

Complete Anti-SARS-CoV-2 IgG seropositivity (100%) and nAb IH% positivity were determined both in patients and controls. A difference with statistical significance in favor of the patients’ group was observed between anti-SARS-CoV-2 IgG titers of two groups (p=0.006), however nAb IH% activity did not differ between two groups (p=0.852). When evaluated in subgroups according to their COVID-19 naïve and recovered status, any significant difference was not also observed between IEM patients and controls in terms of nAb IH% activity while anti-SARS-CoV-2 IgG titers differed significantly (p=0.308 and p=0.017 respectively). Data is shown in **Table 2**.

**Table 2:** Comparison of Anti-SARS-CoV-2 IgG titers and neutralizing antibody IH% activity between patients with inborn errors of metabolism and control subjects after the second dose of BNT162b2 vaccination.

	Patients (n=15)		Controls (n=15)	P value
Anti-SARS-CoV-2 IgG titers, AU/ml	13948.10 (4223.60;40000)		5543.20 (265.10;26225.90)	**0.006**
Neutralizing antibody, % IH	99.3 (94.1;99.6)		99.3 (41.6;99.6)	0.852
	**COVID-19 naïve (n=7)**	**COVID-19 recovered (n=8)**	**Controls (n=15)**	
Anti-SARS-CoV-2 IgG titers, AU/ml	13977.10(10557.60;32999.40)	11967.45(4223.60;40000)	5543.20 (265.10;26225.90)	**0.017**
Neutralizing antibody, % IH	99.2 (97.3;99.4)	99.4 (94.1;99.6)	99.3 (41.6;99.6)	0.308

Data were presented as median (25^th^; 75^th^ percentile). Continuous data were analyzed by the Mann Whitney U test for two groups and the Kruskall Wallis test for three groups. Bold values indicate statistically significant p values (p <0.05).

### Impact of the number of BNT162b2 doses on the humoral immune response in the patient group

Sampling after the first dose of BNT162b2 was available in 12 patients (80%). Among these 12 patients, six were COVID-19 naïve, in whom a complete Anti-SARS-CoV-2 IgG seropositivity (100%) could be obtained. In these patients, the mean antibody titers also significantly increased after the first dose of vaccination (p=0.003). Neutralizing antibody positivity was determined in five COVID-19 naïve patients (83.3%) except one and nAb IH% significantly increased after the first dose of vaccination (p=0.043). In six COVID-19 recovered patients, both Anti-SARS-CoV-2 IgG titers and nAb IH% increased significantly after the first dose of BNT162b2 (p=0.036 and p=0.043, respectively). Data is shown in **Table 3**.

**Table 3:** Evaluation of Anti-SARS-CoV-2 IgG titers and neutralizing antibody IH% activity after the first dose of BNT162b2 vaccination in patients with inborn errors of metabolism.

	Before vaccination	After the 1^st^ dose of vaccination	P value
COVID-19 naïve IEM patients (n=6)			
**Anti-SARS-CoV-2 IgG titers, AU/ml**	0.001	1446.03(656.16)	**0.003**
**Neutralizing antibody, % IH**	0.001	48.8 (0.01;78.2)	**0.043**
COVID-19 recovered IEM patients (n=6)			
**Anti-SARS-CoV-2 IgG titers, AU/ml**	859.73(930.73)	19929.06(16056.86)	**0.036**
**Neutralizing antibody, % IH**	31 (0.001;97.6)	99.5 (0.001;99.6)	**0.043**

Data were presented as mean(SD) or median (25^th^; 75^th^ percentile). Categorical data were analyzed with the Chi-square test or Wilcoxon test where appropriate. Bold values indicate statistically significant p values (p <0.05). IEM, inborn errors of metabolism.

In the first sampling prior to vaccination, the median anti-SARS-CoV-2 IgG titer was 70.70 AU/mL (0.001;2578.10 AU/mL), while it was 13948.10 AU/mL (4223.60;40000 AU/mL) after the second dose of BNT162b2 vaccination. When the mean antibody titers were compared before vaccination and after the second dose of vaccine, a statistically significant increase was observed (p=0.001). Neutralizing antibody concentrations also increased significantly with the second dose of BNT162b2 (p=0.001). When evaluated in subgroups according to their COVID-19 naïve and recovered status, the increase of anti-SARS-CoV-2 IgG titers (p=0.002 and p=0.011 respectively) and nAb IH% activity (p=0.018 and p=0.012 respectively) was also significant in both groups. Data is shown in **Table 4**.

**Table 4:** Evaluation of Anti-SARS-CoV-2 IgG titers and neutralizing antibody IH% activity after the second dose of BNT162b2 vaccination in patients with inborn errors of metabolism.

	Before vaccination	After the 2^nd^ dose of vaccination	P value
IEM patients (n=15)			
**Anti-SARS-CoV-2 IgG titers, AU/ml**	70.70 (0.001;2578.10)	13948.10(4223.60;40000)	**0.001**
**Neutralizing antibody, % IH**	0.001 (0.001;97.66)	99.3 (94.1;99.6)	**0.001**
COVID-19 naïve IEM patients (n=7)			
**Anti-SARS-CoV-2 IgG titers, AU/ml**	0.001	18841.74(9360.58)	**0.002**
**Neutralizing antibody, % IH**	0.001	99.2 (97.3;99.4)	**0.018**
COVID-19 recovered IEM patients (n=8)			
**Anti-SARS-CoV-2 IgG titers, AU/ml**	859.73(930.73)	833.90(788.42)	**0.011**
**Neutralizing antibody, % IH**	36 (0.001;97.66)	99.4 (94.1;99.6)	**0.012**

Data were presented as mean(SD) or median (25th; 75th percentile). Categorical data were analyzed with the Chi-square test or Wilcoxon test where appropriate. Bold values indicate statistically significant p values (p <0.05). IEM, inborn errors of metabolism.

When the anti-SARS-CoV-2 IgG titers and nAb IH% titers after the first and second dose vaccinations were compared, a significant increase continued to be observed in COVID-19 naïve group (p=0.028 and p=0.028, respectively) in contrary to COVID-19 recovered group (p=0.686 and p=0.686, respectively). Data is shown in **Table 5**.

**Table 5:** Evaluation of Anti-SARS-CoV-2 IgG titers and neutralizing antibody IH% activity between the first and second dose of BNT162b2 vaccination in patients with inborn errors of metabolism.

	After the 1^st^ dose of vaccination	After the 2^nd^ dose of vaccination	P value
COVID-19 naïve IEM patients (n=6)			
**Anti-SARS-CoV-2 IgG titers, AU/ml**	1283.50 (663.80;2522)	13962.60 (10557.60;32999.40)	**0.028**
**Neutralizing antibody, % IH**	48.8 (0.01;78.2)	99.2 (97.3;99.4)	**0.028**
COVID-19 recovered IEM patients (n=6)			
**Anti-SARS-CoV-2 IgG titers, AU/ml**	17636.85 (335.80;40000)	14782.10 (4223.60;40000)	0.686
**Neutralizing antibody, % IH**	99.5 (0.001;99.6)	99.4 (94.1;99.6)	0.686

Data were presented as median (25th; 75th percentile). Categorical data were analyzed with the Wilcoxon test. Bold values indicate statistically significant p values (p <0.05). IEM, inborn errors of metabolism.

### Relationship between anti-SARS-CoV-2 IgG and nAb IH % positivity prior to and after BNT162b2 vaccination in the study population

The undetectable titers prior to vaccination in COVID-19 naïve patients can cause a bias when evaluating the correlation between Anti-SARS-CoV-2 IgG and nAb IH% positivity. We were unable to perform a sampling before vaccination in the control group, but it was possible that there were some patients with no prior COVID-19 infection and had undetectable titers. Therefore, both the COVID-19 naïve patients and the control group were excluded from the correlation analysis.

In COVID-19 recovered patients, a strong positive correlation was found between Anti-SARS-CoV-2 Ig G and nAb IH% activity before vaccination (r= 0.934, p =0.001). On the other hand, correlation analysis between Anti-SARS-CoV-2 Ig G and nAb IH% activity in these patients was considered as weak both after the first and second dose of vaccination.

### Clinical efficacy of BNT162b2 vaccination among patients with inborn errors of metabolism

According to the results of the survey conducted six months after the second dose of vaccination, it was learned that none of the patients had post-vaccine COVID-19 infection.

## Discussion

In this prospective study, humoral immune responses to the two-doses of BNT162b2 mRNA COVID-19 vaccine were monitored by assessing anti-SARS-CoV-2 IgG and nAb IH % activity in pediatric IEM patients and compared with healthy controls. Complete Anti-SARS-CoV-2 IgG seropositivity and nAb IH% positivity were observed in all patients after the second vaccination dose, indicating an adequate humoral immune response. Anti-SARS-CoV-2 IgG titers in response to vaccination were significantly increased in IEM patients compared with the control group. Regarding the effects of the number of the BNT162b2 doses, Anti-SARS-CoV-2 IgG titers and nAb IH% increased significantly after the first dose in both COVID-19 naïve and recovered patients. The increase in antibody titers after the first and second vaccination remained significant in COVID-19 naïve patients but did not differ in recovered patients. Vaccination appears to be clinically effective in IEM patients, as none of the patients had COVID-19 infection within six months of the last vaccination.

Vaccination in IEM patients has different considerations in terms of efficacy as immune deficiency can contribute to the course of the disease. Inborn errors of metabolism including disorders of nucleic acid metabolism, cobalamin and folic acid metabolism, congenital disorders of glycosylation, mitochondrial disorders may be associated with primary immune deficiency (8, 9). In addition to these IEM which present as an inborn error of immunity, secondary immune deficiency may be part of the clinical picture in several subtypes due to different pathophysiological mechanisms. Organic acidemia (OA) is the most studied subtype of IEM in terms of secondary immunodeficiency, and it has been suggested that it may result from pancytopenia and/or neutropenia, presumably due to bone marrow suppression by precursory toxic metabolites (19, 20). Hypogammaglobulinemia, depletion of B and/or T-cells, and lack of memory B cell formation despite normal B cell count have also been described even in nonacute phases of the disease (12, 19, 21–24). In lysinuric protein intolerance (LPI), most patients tended to have subnormal IgG subclasses, whereas serum IgG, IgM, and IgA levels were between reference ranges in most patients (11). Elevated lactate levels in the IEM, which are involved in energy production and utilization, contribute to immune suppression through several mechanisms. Because aerobic glycolysis has been shown to play a critical role in immune cell activation, downregulation of glycolytic enzymes by high lactate levels negatively affects the function of immune cells such as T lymphocytes, macrophages and monocytes, dendritic cells, myeloid-derived suppressor cells and natural killer cells (25, 26).

Despite these immune alterations reported in various subtypes of IEM, few studies have been conducted to determine whether this is related to the decreased vaccine response and whether a different vaccination schedule is required to achieve adequate efficacy. Vaccine-specific IgG concentrations have been shown to be a reliable marker for evaluating the humoral immune response after vaccination. In 11 patients with LPI, specific antibody titers against diphtheria were below the assay detection limit in four, against tetanus in three and against conjugated Haemophilus influenzae (Hib) in eight patients. Two patients did not develop adequate response against diphtheria and two against Hib despite booster vaccination. Regarding 23-valent pneumococcus vaccine, six patients failed to develop measurable antibodies against one or more of the three serotypes. (11) In another study, anti-tetanus IgG and anti-diphtheria IgG levels were remarkably low in two patients with maple syrup urine disease (MSUD) despite an appropriate vaccination schedule (12). Most recently, an adequate humoral immune response was reported in ten patients with propionic acidemia (PA) who were vaccinated against measles, mumps, rubella (MMR), and diphtheria/tetanus (DiphtTe) (10). In our study, all IEM patients were found to be completely Anti-SARS-CoV-2 IgG seropositive after the second dose of the vaccination. In addition, the final sampling performed after the second dose of vaccination revealed higher concentrations of vaccine-specific IgG titers compared with controls. However, more comprehensive cohort studies are needed to form a definitive conclusion and to understand the immune response mechanisms in IEM patients against SARS-CoV-2.

Measurement of neutralizing antibody inhibition percentage indicates the functional antibodies capable of inhibiting SARS-CoV-2 infection and appears to correlate with protection by vaccine efficacy (27). It has been suggested that immune protection may decline over time in both vaccinated patients and patients with a history of COVID-19 infection despite measurable anti-SARS-CoV-2 IgG levels. It has been suggested that this decline is related to decreased neutralizing antibody levels and may predict the need for booster vaccinations (28). Adult patients with concomitant diseases including chronic renal failure (hemodialysis and renal transplant), rheumatoid arthritis and solid malignancies have been found to have different neutralizing antibody levels other than healthy individuals, but no data are available for children (29–32). Best of our knowledge, assessment of humoral immune response by evaluating SARS-CoV-2 neutralizing antibodies in IEM patients has not been studied. In our study, nAb IH% positivity was observed in all IEM patients after the second dose of vaccination. Based on the serologic findings, BNT162b was thought to elicit a competent humoral immune response in IEM patients. Depending on the adequate antibody titers observed after the second dose in COVID-19 naïve and even after the first dose in COVID-19 recovered patients, it was assumed that a standard vaccination schedule similar to that of healthy individuals would be sufficient in IEM patients.

As previously mentioned, unvaccinated patients with IEM should be considered a high-risk group with regard to COVID -19 infection, where even life-threatening acute metabolic decompensation may occur (2–6). This study was the first to provide clinical evidence of an adequate immune response, beyond laboratory data, for this particular group for whom the COVID -19 vaccine may be considered mandatory. Because SARS-CoV-2 spike protein-specific memory B cells are thought to be mostly abundant, especially during the first six months after vaccination, our patient group was asked whether they had an active COVID-19 infection within six months after the second dose of BNT162b2 (33). In this present study, results of a survey conducted six months after the second dose of vaccination confirmed the clinical efficacy of vaccination as none of the patients had post-vaccine COVID-19 infection.

Our study had some limitations. The small sample size and uneven distribution of IEM subgroups were due to the very rare occurrence of IEM in the general population and the restriction of patients included in the study to a specific age group. Authors thought that the study had an acceptable sample size compared with studies of vaccine efficacy in pediatric IEM patients. PRNT was not performed to evaluate neutralizing antibody activity in IEM patients. However, we used an antibody test with 98-100% correlation with PRNT in this study. Another limitation was the lack of initial SARS-CoV-2 IgG titers in the control group before vaccination.

In conclusion, humoral immune response after two doses of SARS-CoV-2 mRNA vaccination in pediatric IEM patients appears to be adequate and the immune response is not different from that of healthy individuals. It is recommended that IEM patients be vaccinated against COVID-19 following the regular vaccination schedule to achieve adequate vaccine-specific IgG and nAb titers. Further studies with larger samples will provide information on the immunogenic profile of COVID -19 vaccines in children with IEM.

## Data availability statement

The raw data supporting the conclusions of this article will be made available by the authors, without undue reservation.

## Ethics statement

The studies involving human participants were reviewed and approved by Local Ethical Committee of Istanbul University-Cerrahpasa, Cerrahpasa Medical Faculty. Written informed consent to participate in this study was provided by the participants’ legal guardian/next of kin.

## Author contributions

TZ and HD conceptualized and designed the study, drafted the İnitial manuscript, and critically reviewed and revised the manuscript. DH, RG, EU, GY, SA, GU-I and EO-E designed the data collection instruments, collected data, carried out the initial analyses, and critically reviewed and revised the manuscript. EK designed the data collection instruments, collected data, carried out the initial analyses, and critically reviewed and revised the manuscript. BK and CA-Z conceptualized and designed the study, coordinated, and supervised data collection, and critically reviewed and revised the manuscript for important intellectual content. All authors contributed to the article and approved the submitted version.
